# Effect of Preoperative Continuation of Aspirin on Postoperative Bleeding After Off-Pump Coronary Artery Bypass Graft: A Prospective Cohort Study

**DOI:** 10.7759/cureus.18697

**Published:** 2021-10-12

**Authors:** Muhammad Ishtiaque Al-Manzo, Saikat DasGupta, Sonjoy Biswas, Bappy Basak, Md. Ziaur Rahman, Samir Kumar Biswas, Quamrul Islam Talukder, Prasanta K Chanda, Farooque Ahmed

**Affiliations:** 1 Department of Cardiac Surgery, National Heart Foundation Hospital & Research Institute, Dhaka, BGD; 2 Department of Cardiothoracic Surgery, Square Hospitals Limited, Dhaka, BGD; 3 Department of Cardiac Surgery, United Hospital Limited, Dhaka, BGD; 4 Department of Cardiothoracic Surgery, Liverpool Heart and Chest Hospital, Liverpool, GBR

**Keywords:** postoperative bleeding, preoperative aspirin continuation, coronary artery disease, off-pump cabg, cohort study

## Abstract

Background

Despite ample evidence of continuing preoperative aspirin to improve coronary artery bypass surgery outcomes, practice for the routine continuation of preoperative aspirin is inconsistent due to concern for increased postoperative bleeding. The purpose of this study was to investigate preoperative aspirin use and its effect on postoperative bleeding after off-pump coronary artery bypass grafting (OPCABG).

Methodology

This cohort study involved patients (n = 74) who underwent OPCABG at a single center between August 2017 and January 2018. After considering the inclusion and exclusion criteria, the patients were divided into two groups: one (n = 37) received tablet aspirin 75 mg till the day of the surgery, and for the other group (n = 37) aspirin was stopped five days before the surgery. Postoperative bleeding was recorded in both groups. After considering preoperative, intraoperative, and postoperative variables, statistical analysis was performed.

Results

There was no significant difference between the two groups concerning peroperative and postoperative variables. In addition, no significant difference was observed between the two groups in chest tube drainage at one, two, three, twenty-four, forty-eight, and seventy-two hours (p = 0.845, 0.126, 0.568, 0.478, 0.342, and 0.717, respectively). No significant difference was seen in the transfusion requirement of blood and fresh frozen plasma (FFP).

Conclusions

Continuation of preoperative aspirin till the day of the surgery is neither associated with an increase in chest tube drainage, reoperation for bleeding complications nor transfusion of blood and FFP.

## Introduction

Coronary artery disease (CAD) is among the leading causes of morbidity and mortality worldwide, and its prevalence and adverse impact continue to increase [1]. Coronary artery bypass graft surgery (CABG) is an important therapeutic approach in CAD. Because platelets play a crucial role in the pathogenesis of thrombosis, antiplatelet drugs (especially aspirin) are broadly used as primary, secondary, and tertiary prevention strategies [2]. However, the mechanisms of aspirin that confer protection against myocardial infarction and postoperative graft thrombosis contribute to increased bleeding complications during cardiac surgery. Therefore, it is a long-established practice to stop aspirin a few days before any surgery including CABG. Meanwhile, several studies have demonstrated a reduction of early vein graft thrombosis when aspirin is started soon after CABG. It is associated with a reduction in postoperative ischemia and cardiac, cerebrovascular, gastrointestinal, and renal complications [3]. Furthermore, several studies have reported that preoperative continuation of aspirin does not significantly increase postoperative bleeding after CABG [3-5]. Eventually, the continuation of aspirin before CABG became a recommendation. Despite ample evidence and recommendation regarding continuing aspirin preoperatively before CABG, in Bangladesh, it is not an uncommon practice among surgeons to withhold aspirin before CABG because it is thought to increase postoperative bleeding and related complications [6]. Our study aimed to observe the effect of preoperative aspirin use on postoperative bleeding after off-pump coronary artery bypass grafting (OPCABG), thereby reducing the uncertainty regarding discontinuing aspirin to ensure better patient outcomes. The abstract of this study was presented as a poster at the Association of Surgeons in Training Annual Conference 2021: Excelling in Adversity on March 5-7, 2021.

## Materials and methods

This prospective cohort study involved 74 patients who underwent OPCABG at a tertiary cardiac center between August 2017 and January 2018 to observe the effect of preoperative continuation of aspirin on postoperative bleeding by assessing the amount of bleeding, use of blood products, and incidence of re-exploration. The study protocol was approved by the ethics committee of the institute. All patients admitted under different cardiac surgery consultants for elective CABG were included in the study. Patients on oral or parenteral anticoagulation, or an antiplatelet drug other than aspirin; known allergy or intolerance to aspirin; a history of bleeding diathesis, liver failure, renal failure; and on-pump CABG, redo CABG, or needing other procedures with CABG were excluded from the study. A study of a continuous response variable (amount of postoperative blood loss) from OPCABG with preoperative aspirin and without aspirin was planned. In a previous study [3], the response within each subject group was normally distributed with a standard deviation (SD) of 23.3 mL. As the true difference in the experimental and control means was 15.3 mL, 37 experimental subjects and 37 control subjects were needed to be able to reject the null hypothesis that the population means of the experimental and control groups were equal with a probability (power) of 0.8. The type I error probability associated with this test of the null hypothesis was 0.05. Patients were divided into two groups: one (n = 37) group received tablet aspirin 75 mg till the day of the surgery (Group A), and for the other (n = 37) group, aspirin was stopped five days before the surgery (Group B). After considering preoperative, intraoperative, and postoperative variables, statistical analysis was done by SPSS version 16.00 (SPSS Inc., Chicago, IL, USA). Statistical analysis was done using unpaired t-test between the groups and paired t-test within the groups for normally distributed data and Mann-Whitney U test for data that are not normally distributed. A Chi-square test was done for comparing categorized data. Continuous variables were shown as mean ± SD, and categorical variables were given as a number (percentage). A p-value of <0.05 was considered significant. Boxplots were prepared by the statistical package Minitab 18 developed by the Pennsylvania State University.

## Results

The baseline characteristics of the 74 patients are outlined in Table 1. Most patients were in the 51-60-year age group and were males. According to demography and risk factors, both groups were similar. In addition, both groups did not have a significant difference regarding preoperative hemoglobin level and other coagulation parameters.

**Table 1:** Patient characteristics. Categorical data are presented as n (%) and continuous data as the mean ± SD. BSA: body surface area; HTN: hypertension; DM: diabetes mellitus; BT: bleeding time; CT: clotting time; INR: international normalized ratio

	Group A (Aspirin continued) n = 37	Group B (Aspirin stopped) n = 37	P-value
Age (year)	54.92 ± 7.03	52.62 ± 8.5	0.209
Male	35 (94.6)	35 (94.6)	
BSA (m^2^)	1.70 ± 0.16	1.7 ± 0.12	0.618
HTN	28 (75.7)	30 (81.1)	0.572
DM	16 (43.2)	17 (45.9)	0.815
Ex-smoker	23 (62.2)	20 (54.1)	0.480
Hemoglobin (g/dL)	13.19 ± 1.35	13.00 ± 1.19	0.519
Platelet (100,00/mm^3^)	3.02 ± 0.67	2.97 ± 0.70	0.771
BT (min)	3.69 ± 0.458	3.65 ± 0.44	0.716
CT (min)	5.53 ± 0.53	5.52 ± 0.49	0.973
INR	1.09 ± 0.05	1.08 ± 0.04	0.138

**Figure 1:**
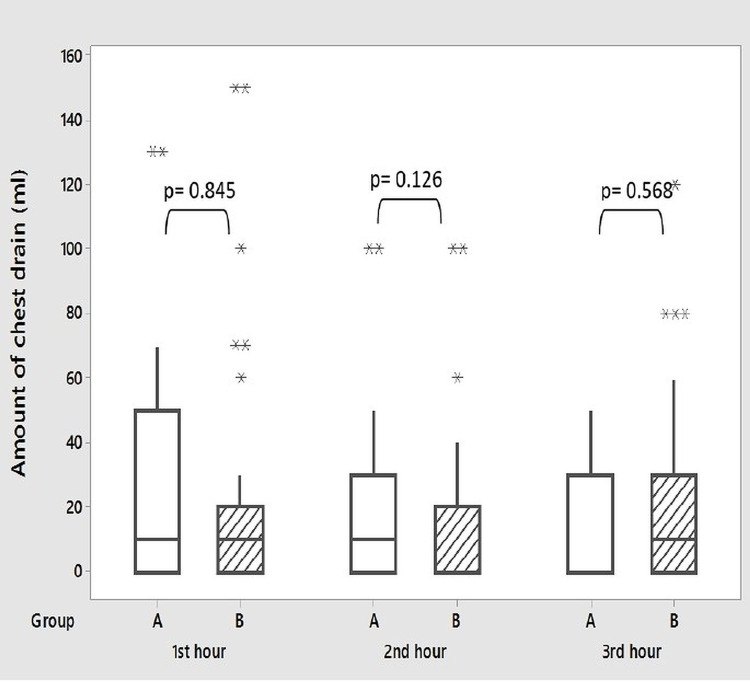
Postoperative chest tube drain in the first three hours. Group A: aspirin continued; Group B: aspirin stopped. Box represents the middle 50% of the data. The line within the box represents the median value. The whiskers represent the ranges for the top 25% of the data, excluding outliers; * represents outliers.

**Figure 2:**
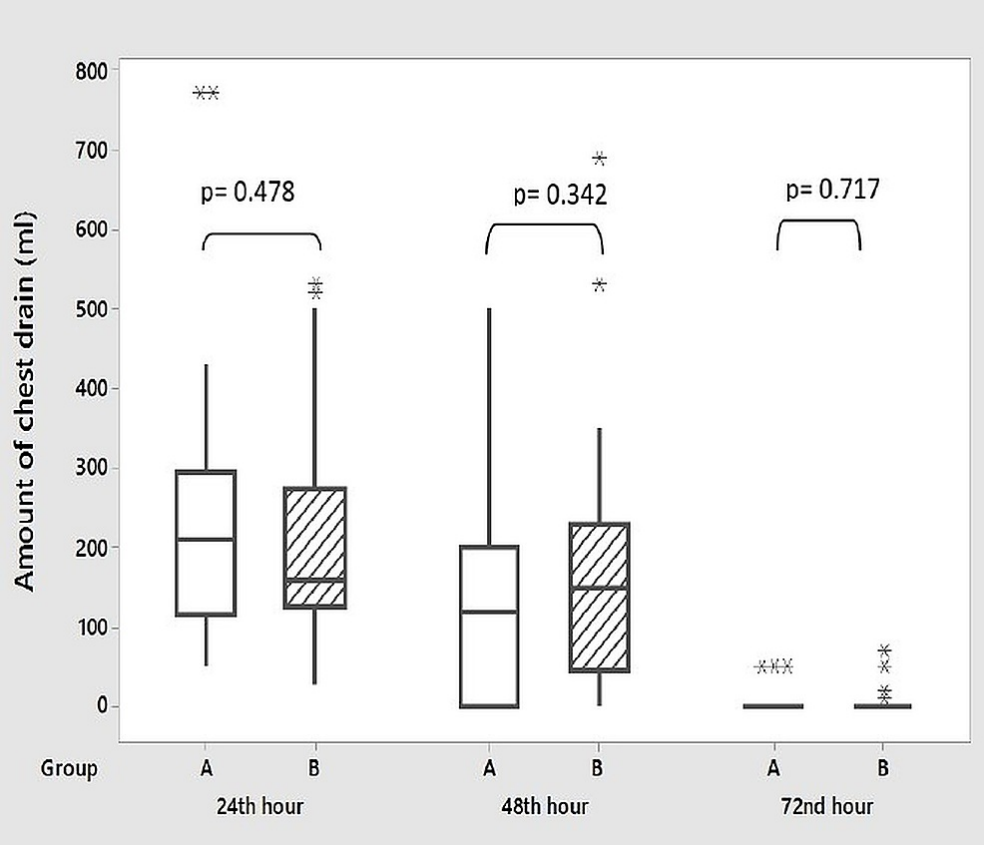
Postoperative chest tube drain at 24, 48, 72 hours. Group A: aspirin continued; Group B: aspirin stopped. Box represents the middle 50% of the data. The line within the box represents the median value. The whiskers represent the ranges for the bottom 25% and the top 25% of the data, excluding outliers; * represents outliers.

**Figure 3:**
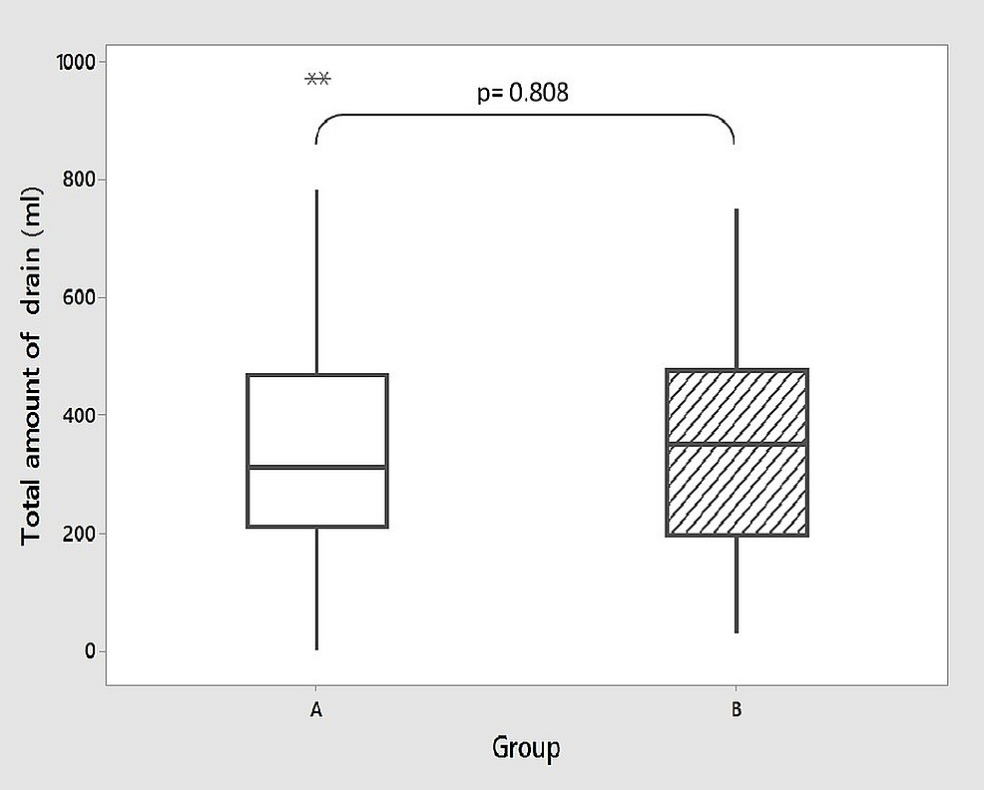
Total amount of postoperative chest tube drain. Group A: aspirin continued; Group B: aspirin stopped. Box represents the middle 50% of the data. The line within the box represents the median value. The whiskers represent the ranges for the bottom 25% and the top 25% of the data, excluding outliers; * represents outliers.

During surgery, transfusion of fresh whole blood (recently collected unrefrigerated blood rich in platelets and coagulation factors) and stored blood (collected from standard blood donation and refrigerated) was 78.4% and 32.4% in the aspirin continued group and 64.9% and 37% in patients who stopped aspirin preoperatively, respectively (p = 0.197 and 0.471, respectively). None of the patients required transfusion of fresh frozen plasma (FFP). The amount of peroperative fluid infusion was also similar in both groups (p = 0.167) (Table 2). Postoperative chest drainage was recorded in both groups at one, two, three, twenty-four, forty-eight, and seventy-two hours (Figures 1, 2). In Group A (n = 37), the median of chest drains was 10 mL, 10 mL, 00 mL, 210 mL, 120 mL, and 0 mL at one, two, three, twenty-four, forty-eight, and seventy-two hours, respectively. In Group B (n = 37), the median of chest drains was 10 mL, 00 mL, 10 mL, 160 mL, 150 mL, and 0 mL, respectively. The median of the total amount of chest drain was 310 mL and 350 mL in Group A and B, respectively (Figure 3). There was no significant difference between the two groups in any of these variables (p = 0.845, 0.126, 0.568, 0.478, 0.342, and 0.717, respectively). No significant difference was also seen in the fluid requirement, transfusion of blood, and FFP postoperatively. None of the patients needed re-exploration for bleeding. Chest drain tube was removed on the second postoperative day (POD) in most of the patients. Haemoglobin, hematocrit, and platelet count were measured on the third POD which showed no significant difference between both groups (Table 3).

**Table 2:** Peroperative transfusion/infusion requirement. Categorical data are presented as n (%) and continuous data as the median (interquartile range).

		Group A (Aspirin continued) n = 37	Group B (Aspirin stopped) n = 37	P-value
Fresh whole blood (units)	0	8 (21.6)	13 (35.1)	0.197
	1	29 (78.4)	24 (64.9)	
Stored blood (units)	0	25 (67.6)	27 (73)	0.471
	1	12 (32.4)	9 (24.3)	
	2	0	1 (2.7)	
Crystalloid (mL)		1,500 (1,500–1,000)	1,200 (1,500–1,000)	0.167

**Table 3:** Postoperative variables. Categorical data are presented as n (%) and continuous data as mean ± SD/median (interquartile range).

		Group A (Aspirin continued) n = 37	Group B (Aspirin stopped) n = 37	P-value
Fresh blood (units)	0	23 (26.2)	27 (73)	0.399
	1	12 (32.4)	7 (18.9)	
	2	2 (5.4)	3 (8.1)	
Whole blood (units)	0	33 (89.2)	29 (78.4)	0.172
	1t	4 (10.8)	8 (21.6)	
Fresh frozen plasma (units)	0	37 (100)	36 (97.3)	0.505
	1	0	1 (2.7)	
Crystalloid (mL)		1,220 (1,390–1,022)	1,150 (1,290–950)	0.081
POD of chest drain tube removal	2nd	26 (70.30)	23 (62.2)	0.542
	3rd	9 (24.3)	12 (32.4)	
	4th	2 (5.4)	1 (2.7)	
	5th	0	1 (2.7)	
Platelet (100,000/mm^3^)		2.68 ± 0.047	2.71 ± 0.67	0.836
Hemoglobin (g/dL)		11.91 ± 1.17	11.84 ± 1.35	0.812
Hematocrit (%)		34.74 ± 3.27	36.00 ± 3.79	0.130

## Discussion

The results of this study support that continuation of preoperative aspirin does not cause increased postoperative bleeding after OPCABG. There was no significant difference between the aspirin continued and aspirin stopped group regarding demographic variables, risk factors, and peroperative and preoperative variables. All parameters of postoperative bleeding, including the amount of chest drainage, blood transfusion, infusion of colloid and crystalloid solutions, and re-exploration for postoperative hemorrhage were similar in both groups.

Platelet dysfunction is one of the major causes of postoperative bleeding after CABG which could be the result of preoperative aspirin continuation but there are several other causes such as inadequate surgical hemostasis, reduced plasma coagulation factor, incomplete heparin reversal, and thrombocytopenia. Accumulating evidence shows that preoperative aspirin continuation does not affect postoperative bleeding significantly after CABG. Kamran et al. (2008) [3] concluded that contrary to commonly held beliefs, the use of aspirin until the date of surgery does not increase the risk of postoperative bleeding after OPCABG. In contrast, they showed a reduction in the bleeding incidence among patients in whom aspirin was not withheld before surgery. However, in our study, the mean of the total amount of chest drain was almost similar, that is, 354.32 mL and 351.08 mL for Group A and Group B, respectively.

Several studies in which preoperative administration of aspirin caused increased postoperative bleeding used a higher dose of aspirin (>150 mg). For example, Sethi et al. (1990) [7] and Ferraris et al. (2002) [8] used 325 mg aspirin once daily. In Bangladesh, the usual practice of aspirin administration is 75-150 mg once daily. At this lower dose, patients are not expected to bleed more postoperatively. Our finding is a reflection of this expectation.

Blood transfusion after surgery is now discouraged due to several adverse effects. However, increased bleeding might increase transfusion requirements. Goldhammer et al. (2015) [9] investigated preoperative aspirin use and its effect on bleeding and transfusion in cardiac surgery. Among those transfused with red blood cells, no significant difference in mean units transfused or massive transfusion was observed. In addition, no significant difference was seen in the transfusion requirement of FFP or platelets. A similar result was found by Kamran et al. (2008) [3] and Wu et al. (2015) [10]. Our study also showed no significant difference between the two groups in transfusion requirement, both during and after surgery. Moreover, the use of cell salvage methods might further reduce the transfusion requirement.

Removal of the chest drain tube is associated with early mobilization, improved respiratory effort, shorter intensive care unit (ICU) stay, reduced postoperative pain, and accelerated overall recovery. The chest drain tube is usually removed on the second or third POD after CABG [3,9]. Most of our patients had their chest tube removed on the second POD, and there was no significant difference regarding the time of chest tube drain removal between the two groups (p = 0.542).

Furthermore, no difference was noted regarding preoperative and postoperative hemoglobin, hematocrit, and platelet count among the two groups. However, both groups showed a significant decrease in all these parameters noted postoperatively in comparison to their preoperative values. A similar observation was noted by Lee et al. 2017 [11].

Surgical re-exploration for bleeding is associated with a significant increase in postoperative mortality as well as an increase in ICU and hospital stay. While Wu et al. (2011) [10] and Jacob et al. (2011) [12] found no increase in re-exploration due to continuation of aspirin preoperatively, Ferraris et al. (2002) [8] and Sethi et al. (1990) [7] observed increased reoperation for bleeding in the aspirin group. None of our patients needed re-exploration for increased bleeding postoperatively.

The use of antifibrinolytic drugs became popular for their role in reducing postoperative bleeding. Pleym et al. (2003) [13] reported that tranexamic acid reduces postoperative bleeding in CABG patients treated with aspirin until the day before the surgery. We used injection tranexamic acid 500 mg intravenously before closing the chest in all our patients.

For ensuring the reduced risk of thrombotic accidents during the peroperative period, Spadaccio et al. (2015) [14] analyzed postoperative bleeding in patients scheduled for elective CABG, depending on the preoperative continuation of aspirin or its replacement with low-molecular-weight heparin (LMWH). The LMWH group was found to bleed more than the aspirin continued group. Hence, they recommended continuing aspirin in preoperative settings.

This study has limitations including smaller sample size, focus only on postoperative bleeding, and purely observational. Furthermore, peroperative blood loss was not measured. As the study population was selected from only one hospital in Dhaka city, the results of the study may not reflect the exact picture of the country.

## Conclusions

The continuation of aspirin throughout the surgery period is not associated with an increase in chest tube drainage, reoperation for postoperative bleeding, or increased requirement of transfusion of blood and blood products. Therefore, our recommendation is to continue aspirin during the perioperative period of OPCABG without the concern of postoperative bleeding and bleeding-related complications.
